# Supplementary material for: Island Hopping through Urban Filters: Anthropogenic Habitats and Colonized Landscapes Alter Morphological and Performance Traits of an Invasive Amphibian
Source: Animals (Basel). 2022 Sep 23;12(19):2549. doi: 10.3390/ani12192549 (PMC9559409; doi:10.3390/ani12192549)
Supplement: Supplementary file 1 [file animals-12-02549-s001.zip › animals-1817844-supplementary.pdf]

Electronic Supplementary Materials for:

Island Hopping through Urban Filters: Anthropogenic Habitats and Colonized Landscapes Alter Morphological and Performance Traits of an Invasive Amphibian

J. Baxter-Gilbert[1,2]*, J. L. Riley[2,3,4], C. Wagener[1], C. Baider[5], F. B. V. Florens[6], P. Kowalski[7], M. Campbell[8], and J. Measey[1]

1 Centre for Invasion Biology, Department of Botany and Zoology, Stellenbosch University, Stellenbosch 7405, South Africa
2 Department of Biology, Mount Allison University, Sackville, NB E4L 1E2, Canada
3 Department of Botany and Zoology, Stellenbosch University, Stellenbosch 7405, South Africa
4 Department of Biology, Dalhousie University, Halifax, NS B3H 4R2, Canada
5 Department of Zoology, University of Oxford, Oxford OX1 4BH, UK
6 The Mauritius Herbarium, Agricultural Services, Ministry of Agro-Industry and Food Security, Réduit 80835, Mauritius
7 Tropical Island Biodiversity, Ecology and Conservation Pole of Research, Faculty of Science, University of Mauritius, Réduit 80837, Mauritius
8 Blue Tide Solutions, Ballito 4391, South Africa
9 Grow Learning Support, Ballito 4391, South Africa
* Correspondence: jbaxtergilbert@mta.ca

Corresponding author: jbaxtergilbert@mta.ca

September 21, 2022

Background

Post-hoc Examination of Potential Trade-offs

Initially, we did not intend to examine potential shifts in trade-offs between performance traits (i.e., escape speed, endurance, and climbing ability). During our analyses, however, we conducted a set of tests to determine whether such trade-offs were present, and, if so, whether these relationships were changing across the invasion route of the guttural toad (*Sclerophrys gutturalis*). As this was not part of the central question driving this research or considered in the research design, we included these findings within the Electronic Supplementary Material.

Overall, we observed that there was a general negative trade-off between escape speed and endurance and between endurance and climbing ability. Our investigation into trade-offs (via correlation) between these performance traits determined that these were not significantly different between natural and urban populations. Similarly, we did not find significantly different correlations between escape speed and endurance between locations (i.e., Durban, Mauritius, and Réunion). Interestingly, it did appear that the trade-off between endurance and climbing ability increased along the guttural toad's invasion pathways—strengthening and becoming more negative from the native (Durban) and then invasive locations (Mauritius and Réunion). This may suggest that the value of an increased ability to climb was favored and promoted during the invasion and may have been achieved at the expense of the toad's endurance capacity. Additional research specifically aimed at quantifying trade-offs between traits along this species' invasion pathway would be beneficial and likely more informative as this was not the primary aim of our work.

Methods

Correlations among Performance Traits

We investigated trade-offs between performance traits (i.e., escape speed, endurance, and climbing ability) by quantifying correlations for each site type (natural and urban) and study location (Durban,

Mauritius, and Réunion) separately. The methodology of this analysis was guided by the structure of our data. Notably, the sample size differed for each of our performance trait datasets. Thus, we predicted values from each performance trait model for a new dataset of 144 observations that equivalently spanned all variables included in each model. Additionally, snout-vent length in this new dataset was randomly selected for each individual based on the minimum and maximum values observed within each study location. We predicted values from our models using the 'predict.merMod' function from the R package 'lme4' (Bates et al. 2015). Using predicted data enabled us to investigate correlations between traits because we had an equal number of observations for each site type/study location, and accounted for dependencies (e.g., repeated measures of the same individual) and additional explanatory variables (e.g., experimental group) within our raw data. Although not an ideal experimental framework to examine trade-offs, this analysis is presented to provide some insight into the correlations between performance traits in guttural toads.

We calculated Spearman rank-order correlations and their corresponding 95% confidence intervals between performance traits using the 'ci_cor' function in the R package 'confintr' (Mayer 2022) and also tested the significance (α = 0.05) of these correlations using the 'cor.test' function in the R package 'stats' (R Core Team 2018). We then used the function 'cocor.indep.groups' from the R package 'cocor' to test for significant differences between species in trait correlations using Fisher's *z* tests (Diedenhofen & Musch 2015). Furthermore, we used a significance or α level of 0.05 for the comparison between natural and urban site types; however, we applied a Bonferroni correction to our significance level for multiple comparisons between study locations (Durban, Mauritius, and Réunion). For these comparisons, our significance or α level was 0.017 (Gotelli and Ellison 2004).

Results

Correlations among Performance Traits

Correlations between performance traits did not significantly differ between urban and natural sites (Table ESM 1a). In general, there was a negative trade-off between escape speed and endurance, as well as climbing and endurance in both urban and natural sites (Table ESM 1a). Notably, there was no strong or significant correlation between escape speed and climbing in either site type (Table ESM 1a).

At all study locations (Durban, Mauritius, and Réunion), correlations between escape speed and endurance were generally weak, negative, and did not differ among locations (Table ESM 1b). Also, correlations between escape speed and climbing were generally weak, largely neutral, and did not differ among locations, thereby reflecting a lack of trade-off (Table ESM 1b). The correlation between endurance and climbing in Durban was weakly positive (Table ESM 1b; Figure ESM 1i). In contrast, on the islands (Mauritius and Réunion), correlations between these traits were negative (Table ESM 1b; Figure ESM 1ii, iii). Although correlations between endurance and climbing significantly differed between Durban and Réunion, none of the other comparisons were significant (Table ESM 1b).

Tables

Table S1. Spearman rank-order correlations and 95% confidence intervals between performance traits (escape speed (m/s); endurance distance (m); climbing ability) of guttural toads (*Sclerophrys gutturalis*) for each **(a)** site type and **(b)** study location. Significant correlations (α = 0.05) and comparisons (α variable; see table) are in bold.

	Escape Speed vs. Endurance	Escape Speed vs. Climbing	Endurance vs. Climbing
(a) Correlations by Site Type			
Natural	**-0.295 (-0.524, 0.008)**	0.180 (-0.036, 0.363)	**-0.521 (-0.705, -0.253)**
Urban	-0.210 (-0.446, 0.058)	0.206 (0.007, 0.382)	**-0.289 (-0.524, -0.057)**
Site Type Comparison (Fisher's *z* test, α = 0.05)			
Natural vs. Urban	z = 0.534, p = 0.594	z = -0.159, p = 0.874	z = -1.646, p =0.100
(b) Correlations by Study Location			
Durban	-0.277 (-0.527, 0.004)	-0.083 (-0.383, 0.190)	0.186 (-0.108, 0.447)
Mauritius	-0.191 (-0.440, 0.097)	-0.105 (-0.372, 0.162)	-0.255 (-0.511, 0.062)
Réunion	-0.227 (-0.462, 0.061)	0.124 (-0.187, 0.404)	**-0.648 (-0.777, -0.465)**
Study Location Comparisons (Fisher's *z* test; α = 0.0167)			
Durban vs. Mauritius	z = -0.432, p = 0.666	z = 0.105, p = 0.916	z = 2.130, p = 0.033
Durban vs. Réunion	z = -0.253, p = 0.800	z = -0.986, p = 0.324	**z = 4.554, p < 0.001**
Mauritius vs. Réunion	z = 0.179, p = 0.858	z = -1.091, p = 0.275	z = 0.659, p = 0.510

Figures

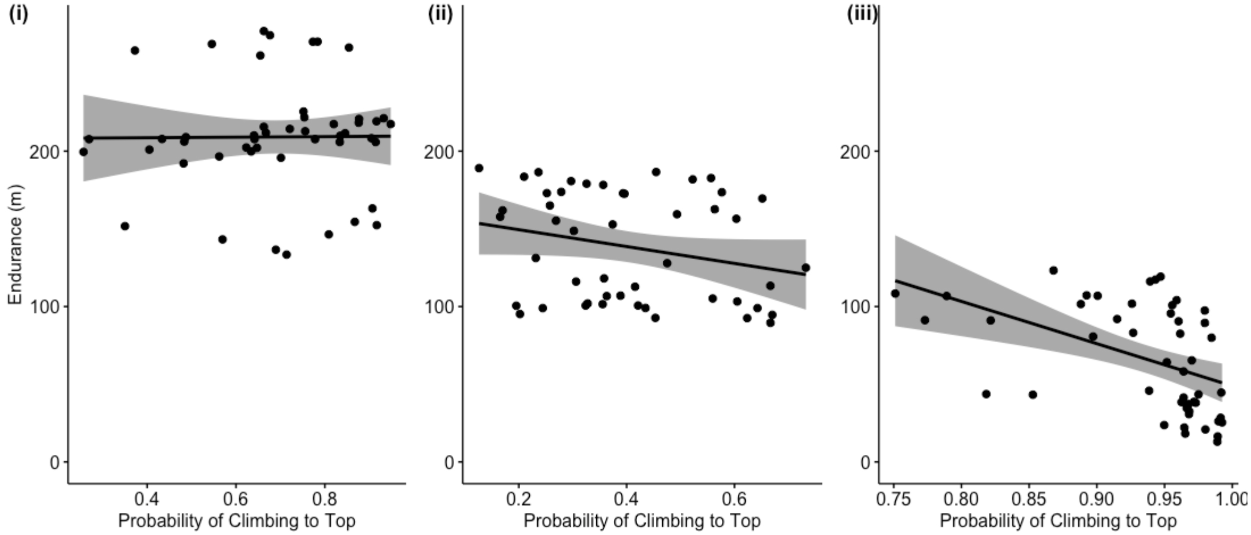

Figure S1. Relationship between guttural toad (*Sclerophrys gutturalis*) endurance (in m) and the likelihood of toads reaching the top of the climbing apparatus (i.e., climbing ability) in **(i)** Durban, **(ii)** Mauritius, and **(iii)** Réunion. These data were predicted from our GLMM, and a line of best fit (in black) overlays the predicted points on the plot. Gray shading around the line of best fit presents the 95% confidence interval. The correlation coefficients significantly differed between Durban and Réunion (R^2_{durban} = 0.186; $R^2_{réunion}$ = -0.648) but not Durban and Mauritius ($R^2_{mauritius}$ = -0.255). Please refer to Table 9 and the main text for more details.

References

Bates, D., Maechler, M., Bolker, B., Walker, S. (2015). Fitting Linear Mixed-Effects Models Using lme4. Journal of Statistical Software, 67(1), 1-48. https://doi.org/10.18637/jss.v067.i01

Diedenhofen, B., & Musch, J. (2015). cocor: A comprehensive solution for the statistical comparison of correlations. PloS one, 10(4), e0121945. https://doi.org/10.1371/journal.pone.0121945

Gotelli, N. J. and Ellison, A. M. (2004) A primer of ecological statistics. Sinauer Associates Inc., Sunderland, MA, USA.

Mayer, M. (2022). confintr: Confidence Intervals. R package version 0.1.2. https://CRAN.R-project.org/package=confintr

R Core Team (2018). R: A language and environment for statistical computing. R Foundation for Statistical Computing, Vienna, Austria. URL https://www.R-project.org/.
